# A single amino acid exchange converts FocA into a unidirectional efflux channel for formate

**DOI:** 10.1099/mic.0.001132

**Published:** 2022-01-27

**Authors:** Michelle Kammel, Oliver Trebbin, Constanze Pinske, R. Gary Sawers

**Affiliations:** ^1^​ Institute for Biology/Microbiology, Martin Luther University Halle-Wittenberg, Kurt-Mothes-Str. 3, 06120 Halle (Saale), Germany; ^†^​ Present address: IMD Laboratory Oderland GmbH, Am Kleistpark 1, Frankfurt (Oder), Germany

**Keywords:** anion channel, conserved histidine, FNT family, formate uptake, hydrophobic pore

## Abstract

During mixed-acid fermentation, *Escherichia coli* initially translocates formate out of the cell, but re-imports it at lower pH. This is performed by FocA, the archetype of the formate-nitrite transporter (FNT) family of pentameric anion channels. Each protomer of FocA has a hydrophobic pore through which formate/formic acid is bidirectionally translocated. It is not understood how the direction of formate/formic acid passage through FocA is controlled by pH. A conserved histidine residue (H209) is located within the translocation pore, suggesting that protonation/deprotonation might be linked to the direction of formate translocation. Using a formate-responsive *lacZ*-based reporter system we monitored changes in formate levels *in vivo* when H209 in FocA was exchanged for either of the non-protonatable amino acids asparagine or glutamine, which occur naturally in some FNTs. These FocA variants (with N or Q) functioned as highly efficient formate efflux channels and the bacteria could neither accumulate formate nor produce hydrogen gas. Therefore, the data in this study suggest that this central histidine residue within the FocA pore is required for pH-dependent formate uptake into *E. coli* cells. We also address why H209 is evolutionarily conserved and provide a physiological rationale for the natural occurrence of N/Q variants of FNT channels.

## Introduction

Formate is a by-product of enterobacterial mixed-acid fermentation and is an energy source for many microorganisms [1–3]. The physiology of formate metabolism is probably best understood for *Escherichia coli *. In *E. coli *, formate is produced from pyruvate by PflB (pyruvate formate-lyase) [4]. Especially under low-pH conditions, formate is further disproportionated to H_2_ and CO_2_ by the cytoplasmically oriented, membrane-associated formate hydrogenlyase (FHL) complex [2, 5]. During fermentation with glucose, and at pH ≥6.5, formate is translocated to the periplasm. However, at a medium pH ≤6.0–6.5, formate/formic acid is re-imported and metabolized by the FHL complex [6]. Bi-directional translocation of formate across the cytoplasmic membrane is carried out by the formate channel, FocA [6]. How FocA controls the direction of passage of formate across the membrane in response to pH is unresolved. Moreover, it is also unknown whether formate, or undissociated formic acid, is translocated through FocA. Uptake of the formate anion into the negatively charged cytoplasm would be thermodynamically challenging, if it acts as a channel for the anion, compared to flow of the undissociated acid down a concentration gradient.

FocA belongs to the family of evolutionarily ancient, pentameric FNT (formate-nitrite transporter) anion channels, which are widespread in bacteria, but are also found in some archaea and protists [7–10]. Each protomer of the pentamer possesses a pore through which small monovalent anions are specifically translocated. Since the discovery of FocA [6], several other FNTs have been identified, which translocate other monovalent anions, such as nitrite (NirC), hydrosulfide (HSC) and lactate (*Pf*FNT) [11–16].

**Fig. 1.**
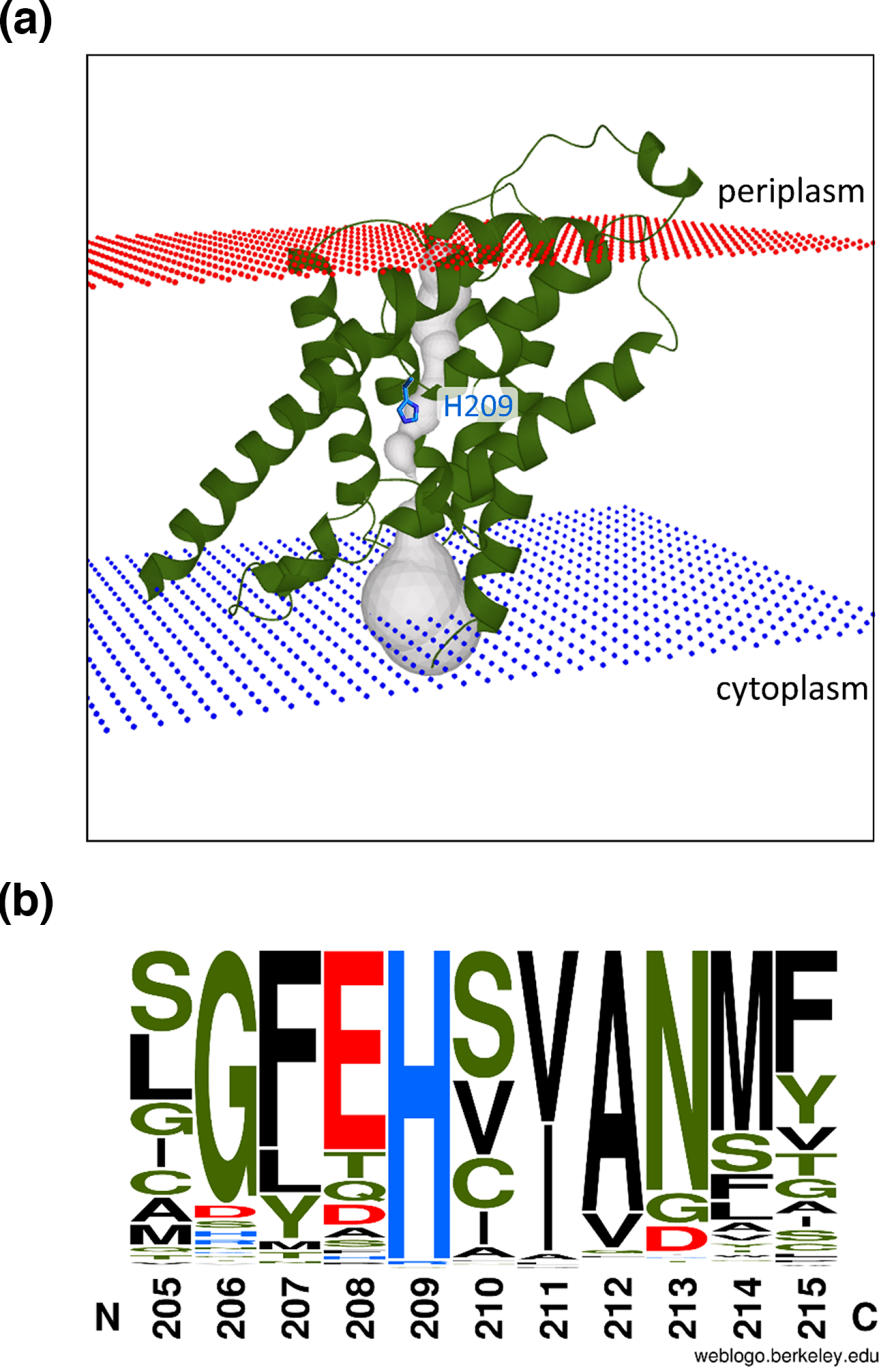
Conservation of histidine residue 209 within the pore of FNT channels. (**a**) Illustration of the central position of histidine 209 (stick representation, blue) within FocA’s translocation pore. The protomer (PDB 3KCU [8]) is displayed in green and ribbon representation and the pore (grey) was modelled using the MOLE 2.5 online tool (for parameters, see Methods). (**b**) Conservation plot comparing the amino acid residues between positions 205 and 215 using 306 FNT channels to create a WebLogo 3 stacking plot (see Methods). The residues are aligned with native FocA from *E. coli *, whose sequence (residues 205–215) is presented below the plot. Amino acid residues displayed in black possess an apolar side chain (**A, F, I, L, M, P, V, W**) and polar side chains are indicated in green (**C, G, N, Q, S, T, Y**). Acidic amino acids are shown in red (**D, E**) and basic amino acid residues are shown in blue (H, K, R).

The translocation pore can be viewed as two funnel-like vestibules connected by a hydrophobic central core (see Fig. 1a). The vestibules are accessible to the cytoplasm and periplasm and abut the hydrophobic core via two narrow constriction sites, which act to restrict anion access [8–10]. Surprisingly, the hydrophobic core of all FNT channels has a similar set of conserved amino acid residues lining the pore [17, 18]. This suggests that FNT channels share a common mechanism for anion translocation and that anion selectivity occurs at the vestibules [17–19]. Evidence supporting this proposal was provided by showing that the soluble cytoplasmic N-terminal domain, which engages with the cytoplasmic vestibule, is important in gating formate translocation by *E. coli* FocA [20]. Nevertheless, as the N-terminal domain controls formate translocation in both the outward and the inward directions, this suggests that a further mechanism determines the pH-dependent direction of formate passage through FocA.

Of the conserved residues within the hydrophobic core of the pore, the only residue that can acquire a charge is a centrally located histidine (H209 in *E. coli* FocA; see Fig. 1a). The histidine is part of an interaction network with a threonine and a bound water [11], which has been suggested to allow H^+^ recycling, and to aid directional control of anion passage through the hydrophobic core of the channel [17, 18]. Notably, H209 is conserved in ~99 % of FNT family members [19], with the exceptions having either asparagine or glutamine at this position (Fig. 1b). The p*K*_a_ of the imidazole side chain of histidine is approximately 6.0, suggesting that this residue might be involved in governing pH-dependent formate passage through the pore. An earlier mutagenesis study showed that exchange of H209 for a tyrosine residue resulted in apparent increased intracellular formate levels, suggesting impaired anion efflux [21]. Electrophysiological studies also implicated H209 as being required for anion translocation [10, 11], whereas a study in which the histidine was replaced by one of the naturally occurring non-protonatable amide amino acids (Gln or Asn) concluded that the histidine has a mainly structural role [19, 22]. To resolve whether the histidine residue has a structural role or a function in anion translocation, we tested these models under physiological conditions and in the homologous host, *E. coli *. We make use of a chromosomally encoded, formate-responsive *fdhF_P_::lacZ* fusion, which enables facile monitoring of changes in intracellular formate concentration [20, 21, 23]. Our findings reveal that exchange of H209 for either asparagine or glutamine converts FocA into an exclusive formate-efflux channel and demonstrate the significance of the cellular context for regulation of formate translocation.

## Methods

### Bacterial strains, plasmids and growth conditions

The strains and plasmids used in this study are listed in Table S1 (available in the online version of this article). The growth conditions used in this study were exactly as described previously [20]. Growth of strains for analysis of β*-*galactosidase enzyme activity, for hypophosphite-sensitivity analysis, for quantitative assessment of growth, for determination of formate, lactate and glucose levels in the culture medium, and for preparation of membrane fractions for subsequent western blot analysis was done anaerobically in M9-minimal medium [24], at 37 °C, and with 0.8 % (w/v) glucose as carbon source. To determine the growth rate under aerobic conditions, the cells were cultivated in 250 ml baffled flasks containing 50 ml M9-minimal medium with 0.4 % (w/v) glucose under vigorous shaking (250 r.p.m.) at 37 °C. To analyse growth of strains in rich medium, TGYEP medium [25], including 1 % (w/v) tryptone, 0.5 % (w/v) yeast extract and 100 mM potassium phosphate pH 7 and 0.8 % (w/v) glucose, was used. Sodium formate was added to a final concentration of 20 mM, where indicated, and antibiotics were used at a final concentration of 100 µg ml^−1^ for kanamycin and 100 µg ml^−1^ for ampicillin, when required.

### Constructions of strains and plasmids

Plasmid pfocA carries the native *E. coli focA* gene and was constructed using pfocA3 [26]. Therefore, pfocA3, a pASK-IBA3 plasmid coding for *focA* with a C-terminal StrepII-tag, was used as template and a stop-codon was re-introduced at the 3′ end of the gene, truncating the StrepII-fusion peptide. The oligonucleotide primers (IDT BVBA) used for this site-directed mutagenesis experiment were focA_stop_fw and focA_stop_rev and are listed in Table S2. All mutated *focA* genes encoding the amino acid exchange variants of FocA used in this study were constructed with pfocA as DNA template and the oligonucleotide primers listed in Table S2, with site-directed mutagenesis experiments carried out according to the Agilent QuikChange protocol (Agilent Technologies). To introduce a chromosomal substitution of codon 209 in the *focA* gene, a 2220 bp fragment, ranging from ∼460 bp upstream of *focA* to codon 280 within *pflB*, was amplified by PCR with oligonucleotides focA_pMAK_fw and focA_pMAK_rev (Table S2) using MC4100 genomic DNA as template. This PCR fragment, which included 5′ *Hind*III and 3*′ Sac*I restriction sites, was first subcloned into pJET1.2 (Thermo Fischer). Site-directed mutagenesis to replace histidine 209 with asparagine was performed as described above (oligonucleotides in Table S2). The *focA-pflB* DNA fragment, including the codon 209 substitution, was excised from pJET1.2 by digestion with *Hind*III and *Sac*I and cloned into the similarly digested pMAK705 [27]. The mutated *focA* allele was then recombined into the chromosome of strain MC4100, following the protocol of Hamilton *et al*. [27], and resulted in strain MC4200.

To introduce the formate-responsive *lacZ*-based reporter, MC4200 and REK702 were transfected with phage *λ(fdhF_P_::lacZ*) [23], yielding strains DH4200 and DH702, respectively. All introduced site-specific mutations were verified by DNA sequence analysis of the complete *focA* gene.

### Preparation of membrane fractions and protein purification

Preparation of membrane fractions for the identification of FocA variants was performed exactly as previously described [20].

### PAGE and immunoblotting

Western blot analysis of membrane fractions bearing FocA, in which polypeptides were separated by gel electrophoresis using either 12.5 % (w/v) SDS gels [28] or 16 % Tris-tricine gels [29], was carried out using anti-FocA antiserum (typically diluted 1 : 1000) as described [20, 30].

### Hypophosphite-sensitivity test

The sensitivity of strains transformed with plasmids encoding various FocA variants toward the formate analogue hypophosphite was tested by determining anaerobic growth rates of strains at 37 °C in M9 minimal medium containing 0.8 % (w/v) glucose and 0.5 mM sodium hypophosphite, as previously described [20]. Experiments were performed in triplicate and growth rates are presented with standard deviation of the mean.

### β-Galactosidase enzyme activity assay

The β-galactosidase enzyme activity was determined and calculated according to Miller [31], but performed with the modifications described [20].

### Analysis of formate, lactate and glucose concentrations in the culture medium

The analysis of extracellular formate and glucose levels by HPLC was performed exactly as described [20]. The concentration of organic acids in the culture supernatant was monitored at 210 nm and glucose concentration was determined using the refractive index detector.

### Analysis of formate translocation and metabolic changes through the growth phase

To analyse formate translocation over time with regard to growth and pH of the culture medium, strains were grown in serum bottles (100 ml cultures in M9-minimal medium with glucose as carbon source), and samples were taken every hour and used for β-galactosidase enzyme activity analysis. The respective supernatants were then used to determine organic acid and glucose levels by HPLC and to determine pH. The experiments were performed with minimally three biological replicates and the determined parameters are presented with standard deviation of the mean. To analyse the effect of elevated FocA levels on translocation activity, *focA* expression was induced by addition of AHT (anhydrotetracycline) at 0.2 μg ml^−1^, when cells entered the exponential growth phase (OD_600_ ~0.4).

### Analysis of H_2_ production via GC

An additional analysis to yield indirect information on intracellular formate levels is the investigation of FHL complex activity by quantification of the amount of H_2_ production by cultures. DH701 strains complemented with plasmids carrying genes encoding different FocA variants were grown anaerobically in 15 ml Hungate tubes in M9-minimal medium containing glucose with 10 ml head space at 37 °C until cultures reached the end of the exponential growth phase (OD_600_ of 0.6–0.9). GC measurements were carried out as described previously [32]. The experiments were performed with three biological replicates, with each assay performed in duplicate, and the amount of H_2_ was calculated with reference to the optical density (OD_600_) and is presented with standard deviation of the mean.

### Computational tools

To illustrate the central position of His209 within the FocA pore, the MOLE 2.5 online tool [33] was used. A FocA monomer (chain A) of PDB structure 3KCU [8] was used to model the pore using 5 Å as the probe radius and 0.8 Å as the interior threshold. Moreover, the membrane region was visualized.

The degree of conservation of amino acids 205–215 (* E. coli* FocA numbering) of a sequence alignment of 306 annotated FNTs was assessed and displayed using the WebLogo tool (Fig. 1b; online version WebLogo 3) [34].

## Results

### Changing H209 to either Asn or Gln converts FocA to a formate efflux channel

**Fig. 2.**
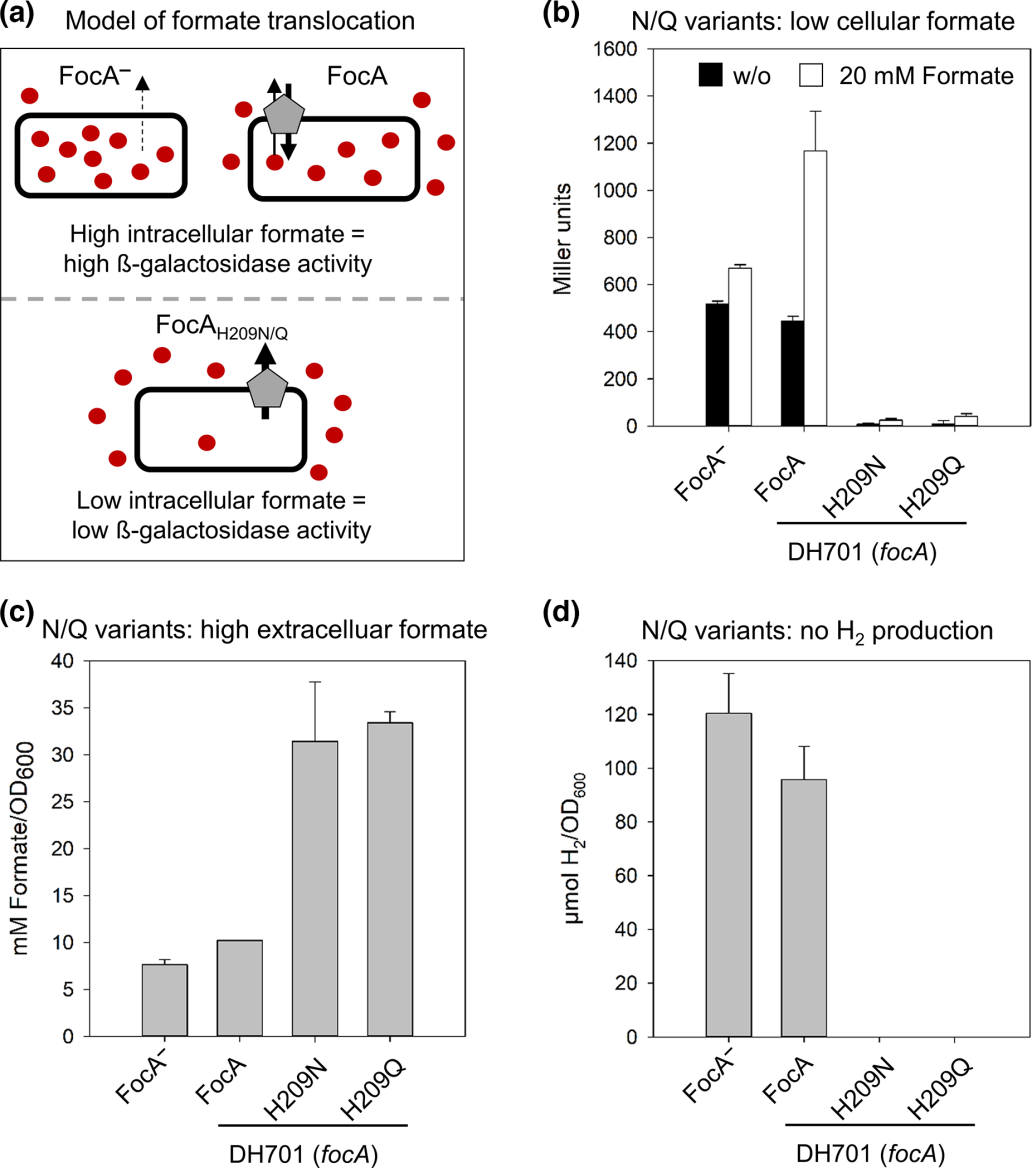
Histidine-209 is essential for bi-directional translocation of formate by FocA. Parameters determined to monitor changes in formate translocation were investigated in the *focA* mutant DH701 (*focA*) and in DH701 complemented with plasmids carrying a *focA* gene encoding the indicated H209 variants. Strains were grown anaerobically in glucose-M9-minimal medium. (a) Schematic representation of the output of the *fdhF_P_::lacZ* reporter system used to monitor changes in intracellular formate (red circles) levels. (b) β-Galactosidase enzyme activity of the respective strains was analysed in cells grown in culture medium without (black histogram) and with exogenously added 20 mM formate (white histogram). (c) The concentration of formate in the culture medium was determined by HPLC analysis. (d) H_2_ evolved by cells accumulated in the headspace of the cultures during growth in glucose-M9-minimal medium. H_2_ was determined quantitatively using GC. All experiments were performed with minimally three biological replicates, with each assay performed in duplicate.

To determine the importance of H209 for formate translocation (note that unless specifically discussed, we do not distinguish at this juncture between formate or formic acid), we utilized an established *in vivo* formate-responsive reporter system [23], which functions as depicted schematically in Fig. 2(a). This system makes use of a chromosomally localized, formate-sensitive, *lacZ*-based reporter (*fdhF_P_::lacZ*), in which the promoter of the formate dehydrogenase (*fdhF*) gene is used. Strains unable to generate formate intracellulary do not express the *fdhF_P_::lacZ* gene fusion, and consequently have essentially no β-galactosidase enzyme activity [20, 23]. In contrast, an *E. coli focA* mutant (strain DH701; see Table S1) that generates and retains an increased concentration of formate within its cytoplasm after fermentative growth on glucose has a β-galactosidase enzyme activity of 517±13 Miller units (Fig. 2b). As established before [20], introduction of the *focA* gene encoding native, unmodified FocA on plasmid pfocA into strain DH701 resulted in a β-galactosidase enzyme activity that was roughly 85 % of the level measured in DH701 without FocA (Fig. 2b). This control confirms that FocA is able to translocate formate out of the cytoplasm (Fig. 2b). This reporter system thus allows FocA channel activity to be monitored in its physiological context, enabling us to define the role of the H209 residue within the translocation pore. Therefore, we exchanged H209 with either an asparagine (FocA_H209N_) or a glutamine (FocA_H209Q_) residue at this position. These are the only residues known to be substituted naturally for His within FNT channels [19], and, although polar, neither amino acid side-chain can be protonated. Plasmids carrying *focA* genes encoding FocA_H209N_ or FocA_H209Q_ were introduced into strain DH701. Western blotting experiments using anti-FocA peptide antibodies confirmed that these FocA variants were synthesized and correctly located in the membrane fraction of the respective strains (Fig. S1a, b). Fermentative growth, which results in the convertion of up to one-third of the carbon from glucose into formate, failed to show measurable β-galactosidase enzyme activity in strains synthesizing these variants (<10 Miller units, Fig. 2b). These results indicate that intracellular formate levels in these strains remained below the threshold (approximately 5 mM) required to induce expression of the *fdhF_p_::lacZ* reporter [23, 35]. These FocA_H209N_ and FocA_H209Q_ variants are thus suggested to remove formate with high efficiency from the cell’s cytoplasm.

To address this suggestion, we determined the extracellular formate concentration in the culture medium for strain DH701 synthesizing either FocA_H209N_ or FocA_H209Q_ (Fig. 2c). When strain DH701 synthesizes either of these two variants, extracellular formate accumulated to a concentration of approximately 30 mM OD_600_^−1^, which is a concentration roughly three times higher than in cultures containing cells synthesizing the native FocA channel (10.2 mM OD_600_^−1^, Fig. 2c). This experiment confirmed that strain DH701 synthesizing FocA_H209N_ or FocA_H209Q_ translocated more formate into the extracellular medium compared to the native FocA protein with a histidine residue at amino acid position 209.

Next, we tested whether adding 20 mM formate to the medium from the start of growth could overcome the lack of expression of the *fdhF_P_::lacZ* fusion in strains synthesizing FocA_H209N_ or FocA_H209Q_. Exogenously added formate (20 mM) failed to cause any apparent increase in the β-galactosidase enzyme activity of strain DH701 synthesizing either FocA_H209N_ or FocA_H209Q_ (<50 Miller units, Fig. 2b), while addition of 20 mM formate to a culture of strain DH701 synthesizing native FocA caused an approximate 2.5-fold increase in β-galactosidase enzyme activity (>1100 Miller units, Fig. 2b) compared to when no additional formate was added. Thus, exogenously added formate caused accumulation of a higher intracellular formate concentration in cells synthesizing native FocA, compared with that produced from glucose alone; however, strains synthesizing FocA variants with an amide residue replacing H209 were either unable to take up this additional formate or to retain it in the cell. Remarkably, any formic acid that diffused across the cytoplasmic membrane (p*K*_a_ of formate/formic acid=3.75) into cells was also translocated out by FocA_H209N_ or FocA_H209Q_, because these cells had a considerably lower β-galactosidase enzyme activity than strain DH701 lacking any FocA (Fig. 2b). Together, these findings suggest that FocA channels with an asparagine or glutamine residue instead of H209 in the pore immediately translocate formate from the cell and fail to take up formate.

### Cells synthesizing FocA_H209N_ do not evolve H_2_

As a final proof that formate failed to accumulate in cells synthesizing FocA_H209N_ or FocA_H209Q_, we determined H_2_ production after fermentative growth. H_2_ is produced from formate by the FHL complex, whose synthesis requires accumulation of intracellular formate to low millimolar concentrations in order to induce expression of the corresponding structural genes, e.g. *fdhF* [3, 35]. Therefore, H_2_ evolution was monitored in cultures of DH701 (*focA*) synthesizing either native FocA, FocA_H209N_ or FocA_H209Q_ after growth to the late exponential phase, and this was compared to levels of H_2_ evolution in DH701 cells, which do not synthesize FocA (Fig. 2d). The results clearly demonstrated that the strain with FocA_H209N_ or FocA_H209Q_ showed no detectable H_2_ production during glucose fermentation, while the other two strains (DH701 and DH701/pfocA) produced more than 90 µmol of H_2_ OD_600_^−1^. Together, these findings confirm that the FocA_H209N_ or FocA_H209Q_ variant allows strains to maintain an intracellular formate concentration below the threshold necessary to induce *fdhF_P_::lacZ* expression, and concomitantly FHL complex synthesis, at least until the late exponential phase.

### FocA_H209N_ prevents intracellular formate accumulation throughout growth

**Fig. 3.**
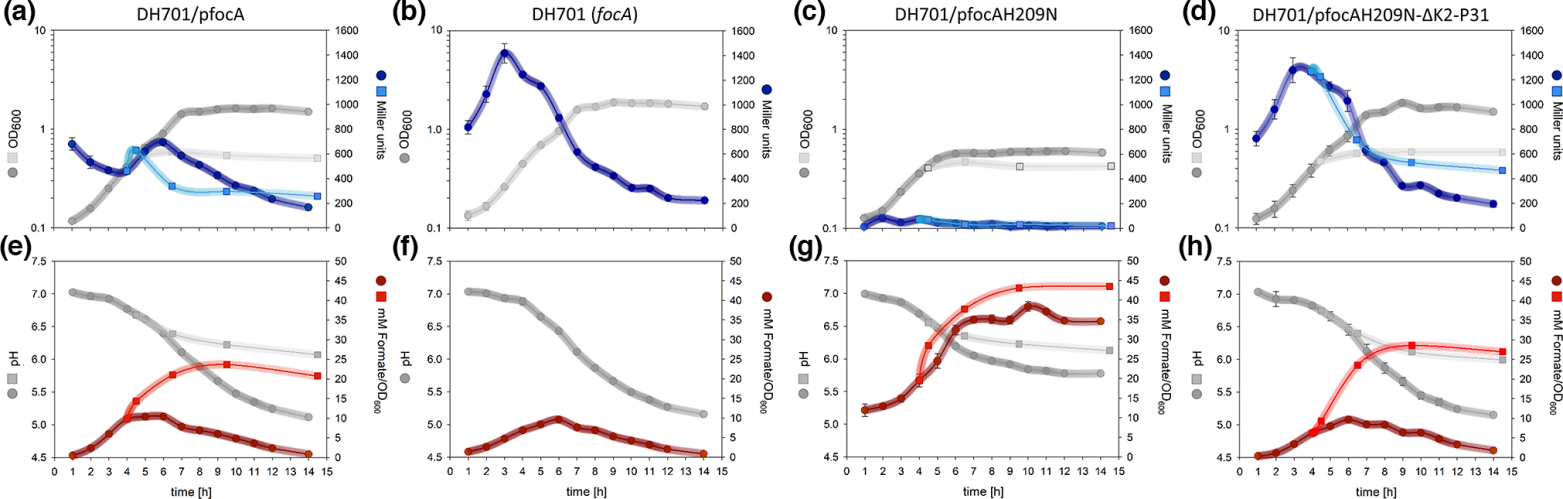
Cells synthesizing the FocA_H209N_ variant function as unidirectional formate efflux channels. Strains DH701 (*focA*), DH701/pfocA, DH701/pfocAH209N and DH701/pfocAH209N-∆K2-P31 were grown anaerobically in glucose-M9-minimal medium in crimp-sealed serum bottles. Samples were taken every hour to analyse optical density (OD_600_), determine β-galactosidase enzyme activity, pH and formate levels of the culture medium. (a, h) Results obtained for strain DH701, (b, f) for strain DH701/pfocA, (c, g) for strain DH701/pfocAH209N and (d, h) for strain DH701/pfocAH209N-∆K2-P31. Parameters obtained for strains, whose *focA* gene expression was elevated by addition of 0.2 mg l^−1^ AHT (anhydrotetracycline) after entering the exponential growth phase, are presented as filled squares. Data for strains grown without AHT treatment are displayed as circles. The colour-coding is as follows: light grey, OD_600_; blue, β-galactosidase activity; dark grey, pH; and red, external formate concentration, which was calculated with respect to OD_600_. All experiments were performed with three biological replicates.

In growing, fermenting *E. coli *, formate is initially translocated out of the cell, but towards the late exponential growth phase the anion is re-imported as a substrate for the FHL complex [6, 23]. To assess how the FocA_H209N_ variant impacts intra- and extracellular formate levels during anaerobic batch-culture growth, we simultaneously monitored expression of *fdhF_P_::lacZ* as β-galactosidase enzyme activity, determined extracellular formate levels and measured the pH of the culture medium (Fig. 3). We also monitored glucose consumption and lactate production (Fig. S3). In strain DH701 synthesizing native FocA, β-galactosidase enzyme activity already registered a level of approximately 700 Miller units after 1 h of growth (Fig. 3a). This initially high level of *lacZ* expression was presumably due to extant gene expression from the stationary-phase culture used for inoculation, coupled with calculations including the low cell density at the beginning of growth. As growth proceeded, the β-galactosidase activity slowly decreased until the mid-exponential phase of growth, where it then increased steadily peaking after approximately 6 h. This indicated production and accumulation of formate within these cells, which induced gene expression and hence increased enzyme activity. After 6 h, β-galactosidase enzyme activity steadily decreased for the next ~6 h, until a 3-fold lower level than that in the late-exponential phase of growth was measured (Fig. 3a). Maximal *lacZ* expression levels, along with the subsequent corresponding shift to lower β-galactosidase enzyme activity, occurred when the pH of the culture medium was approximately 6.5 (Fig. 3e). Analysis of extracellular formate levels (Fig. 3e) in the culture medium revealed a peak at around 5–6 h, and, thereafter, formate levels began slowly to decrease as formate was re-imported into the cells. This disappearance of formate from the culture medium correlated with the reduction in intracellular β-galactosidase enzyme activity and was due to formate consumption by the FHL complex [1, 6].

Glucose was completely consumed by strain DH701/pfocA after 7–8 h, correlating with entry into the stationary phase (Fig. S3a). Additionally, we monitored the extracellular concentrations of other organic acids produced during mixed-acid fermentation, as exemplified for lactate, which maintained a continuous, steady increase in concentration throughout growth (Fig S3a). Whereas the changes in formate levels were dependent on pH and the growth phase and further metabolism, lactate (p*K*_a_=3.86) levels were not.

When the same experiment was repeated with strain DH701 lacking any FocA, it was immediately evident that *fdhF_P_::lacZ* expression started from a similarly high level, as for DH701 synthesizing native FocA, but β-galactosidase enzyme activity continued to increase to much higher levels by the time cells reached the mid-exponential phase of growth (Fig. 3b; approx. 3 h). This indicated accumulation of intracellular formate. Moreover, the kinetics of *fdhF_P_::lacZ* expression were markedly different, particularly because the peak of β-galactosidase enzyme activity was attained earlier in the growth phase and the decrease in *fdhF_P_::lacZ* expression began at a pH of 6.9 (Fig. 3b). Formate accumulation in the growth medium peaked around 6 h (Fig. 3f). Nevertheless, even in the absence of FocA, all of the formate ultimately was taken up by the cell (Fig. 3f). Glucose utilization was similar in DH701 to that in DH701/pfocA (Fig. S3b). Together, these findings suggest a FocA-independent mechanism exists for formate efflux and uptake in a strain lacking FocA, compared to when FocA is present.

A very different *fdhF_P_::lacZ* expression profile was observed during growth of DH701 synthesizing FocA_H209N_ (Fig. 3c). Only very low β-galactosidase enzyme activity could be measured throughout the growth phase, and this was at least 10-fold lower than when DH701 synthesized native FocA (compare Fig. 3c with 3a). Formate was translocated into the growth medium, but was not taken up into the cells, instead accumulating extracellularly to levels that were more than 4-fold higher (with respect to OD_600_) than for cells synthesizing the native FocA protein (Fig. 3g). It was noted that the final cell density of DH701 synthesizing FocA_H209N_ was significantly lower (~35 % reduction) (Fig. 3c) than the parental strain DH701 (Fig. 3b). Despite the decrease in pH of the culture medium below 6.5 after 4 h, no formate uptake by the strain synthesizing FocA_H209N_ occurred.

Analysis of glucose utilization and lactate production by DH701 synthesizing FocA_H209N_ revealed similar kinetics for both metabolites, compared with the parental strain (Fig. S3c). Glucose was, however, not completely consumed by DH701/FocA_H209N_, while lactate production started slightly earlier compared with DH701 or DH701 synthesizing native FocA.

**Fig. 4.**
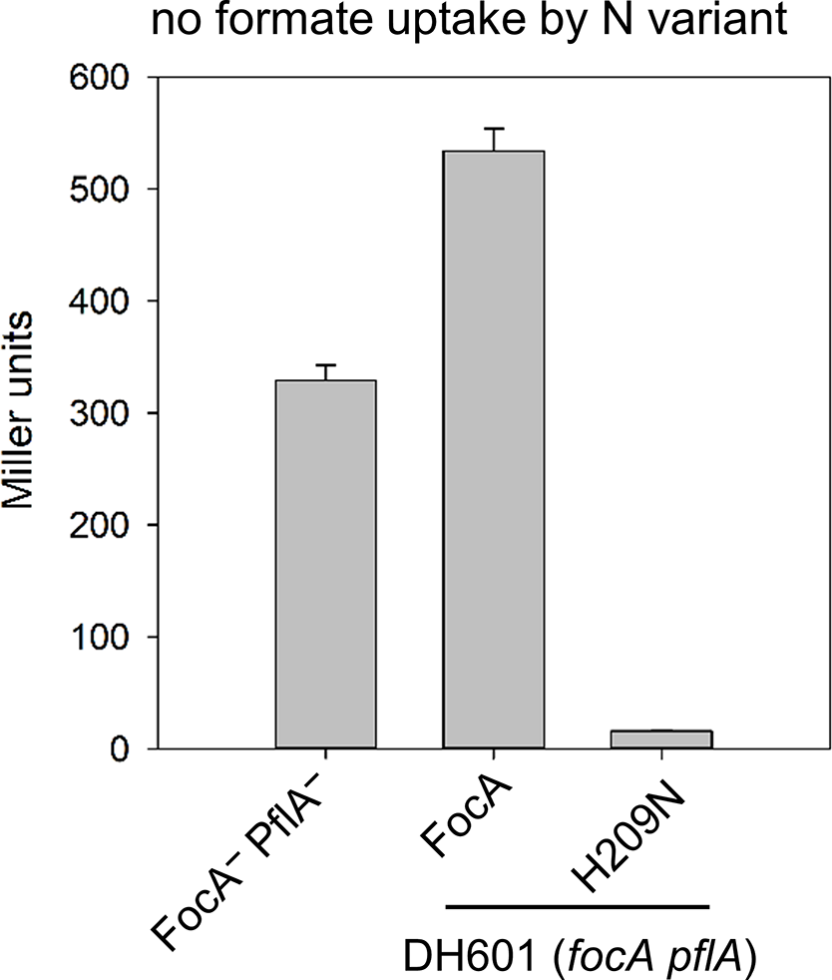
The FocA_H209N_ variant is not able to take up formate. Formate translocation was investigated in the *focA* and *pflA* mutant DH601 (*focA pflA*) and in DH601 complemented with plasmids carrying a gene encoding either native FocA or FocA_H209N_. β-Galactosidase enzyme activity of the respective strains was analysed in cells grown anaerobically in glucose-M9-minimal medium with exogenously added 20 mM formate. The experiment was carried out with minimally three biological replicates, with each assay performed in duplicate.

To demonstrate that FocA_H209N_ does not take up formate, we introduced plasmid pfocAH209N into strain DH601 [20], which cannot activate PflB and consequently fails to make formate intracellularly. After anaerobic growth in glucose-minimal medium supplemented with 20 mM formate, essentially only a very low β-galactosidase enzyme activity could be measured for FocA_H209N_, while native FocA synthesized in the same strain induced an ~33 fold higher β-galactosidase enzyme activity (Fig. 4). This result suggests strongly that the FocA variant with the H209N-exchange failed to import formate into anaerobic *E. coli* cells and that any formic acid entering the cell by passive diffusion was immediately translocated out of the cytoplasm by FocA_H209N_.

### Strain DH701 synthesizing FocA_H209N_ is impaired in import of hypophosphite

**Fig. 5.**
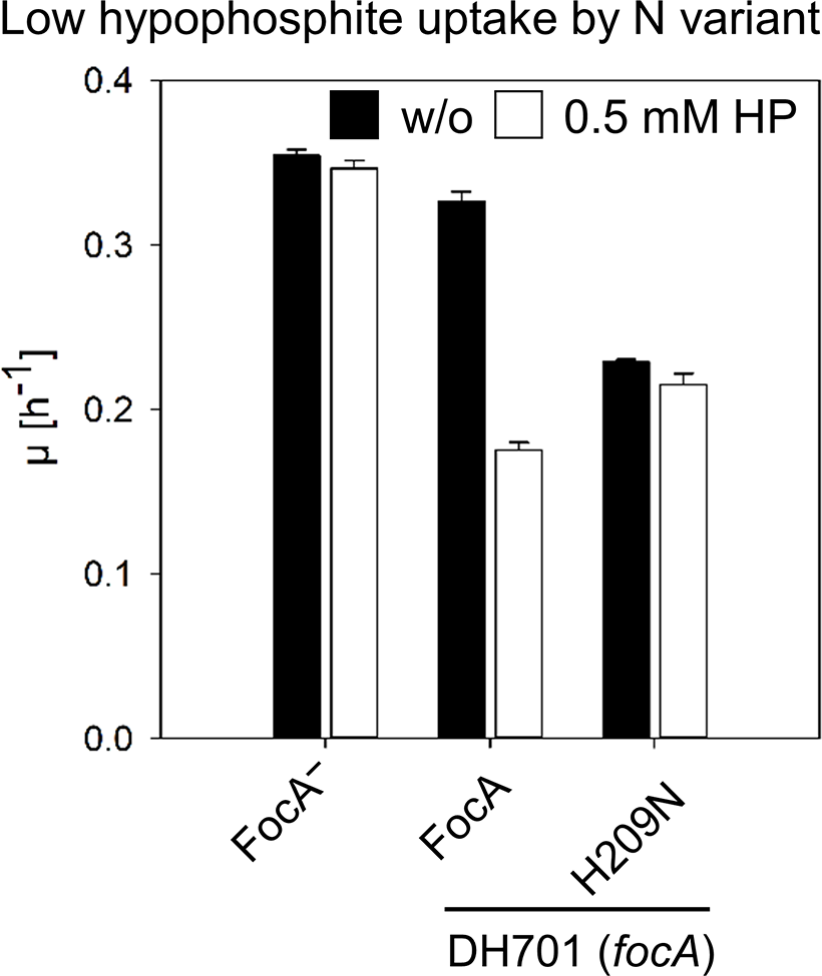
Histidine-209 is essential for uptake of hypophosphite by FocA. Sensitivity of strain DH701 (*focA*) synthesizing different FocA amino acid exchange variants toward hypophosphite was assessed by determining anaerobic growth rates in glucose-minimal medium in the absence (black histograms) or presence of 0.5 mM sodium hypophosphite (white histograms). The experiments were performed in triplicate.

The toxic formate analogue hypophosphite is strongly reducing, and with a p*K*_a_=1.1 cannot be readily taken up by cells by passive diffusion. Like formate, however, hypophosphite can be taken up into *E. coli* cells by FocA, and causes impaired anaerobic growth by inactivating PflB [4, 6, 26, 36]. Therefore, determination of the hypophosphite-sensitive growth phenotype indirectly indicates whether hypophosphite can be imported by FocA variants, because mutants lacking FocA, or uptake-inactivated FocA variants, show a hypophosphite-resistant phenotype [6, 20]. The reduction in growth rate attained after fermentative growth of DH701 (*focA*) upon addition of 0.5 mM hypophosphite was negligible (~2 %) compared with growth in the absence of hypophosphite. This indicates an inability to import hypophosphite due to lack of FocA (Fig. 5). Introduction of the native *focA* gene on plasmid pfocA into DH701 restored the hypophosphite-sensitivity phenotype, whereby the growth rate was reduced by approximately 46 %, indicating FocA-dependent hypophosphite uptake (Fig. 5). In contrast, and compared to growth in the absence of hypophosphite, only a low further hypophosphite-dependent reduction (6 %) in growth rate of strain DH701 synthesizing FocA_H209N_ was observed (Fig. 5). The growth rate of the strain synthesizing FocA_H209N_ in the presence of hypophosphite was nevertheless still higher than that synthesizing native FocA. These findings demonstrate that uptake of hypophosphite by FocA_H209N_ is strongly impaired and fails to result in toxicity to the cells.

### Formate efflux by a strain synthesizing the FocA_H209N_ variant depends on the N-terminal domain

It was recently shown that the soluble, cytoplasmic N*-*terminal domain of FocA is essential for effective channel function [20]. To determine whether the N-terminal domain is also important for the formate efflux function of the FocA_H209N_ variant, a new *focA* gene variant was constructed in which the coding sequence for the N-terminal domain was deleted, along with the altered codon-209 within the *focA* gene (see Methods). Introduction of plasmid pfocAH209N-ΔK2-P31 into DH701 and analysis of *fdhF_P_::lacZ* gene expression during fermentative growth with glucose showed that the strain accumulated formate within the cell, resulting in a high β-galactosidase enzyme activity of 1300 Miller units in the late exponential phase (Fig. 3d). This activity was comparable to that obtained for strains DH701 (lacking FocA) and DH701/pfocAΔK2-P31 (Fig. S2). This contrasts sharply with the barely detectable β-galactosidase enzyme activity throughout the growth phase when FocA_H209N_ was synthesized in strain DH701 (compare Fig. 3c).

Removal of the N-terminal domain of FocA causes some destabilization of the pentamer in the membrane, which results in less protein being detectable [20]. To ensure that sufficient FocA_H209N-ΔK2-P31_ was synthesized in DH701, the experiment was repeated with supplementation of AHT to the growth medium when cells entered the early exponential growth phase. AHT induced expression of the cloned *focA* gene on pfocAH209N-ΔK2-P31 (Fig. 3d, compare Fig. S1c). The profile of β-galactosidase enzyme activity was similar to that after growth without AHT addition, with the exception that activity remained at around 400 units well into the stationary phase. Supplementation of AHT to the DH701/pfocAH209N culture had no effect on β-galactosidase enzyme activity, which remained very low throughout growth (Fig. 3c). Analysis of formate levels in the medium during growth of DH701/pfocAH209N-ΔK2-P31 without AHT addition revealed that the formate concentration was considerably lower than for strain DH701 synthesizing FocA_H209N_ (Fig. 3h). While addition of AHT increased the level of excreted formate in strain DH701/pfocAH209N-ΔK2-P31 to a level comparable to that of DH701 synthesizing native FocA (compare Fig. 3f, h), it also stressed the cells, as reflected by the significantly poorer growth. The excreted formate level was approximately 35 % lower than the level excreted by DH701 synthesizing FocA_H209N_ (compare Fig. 3f with 3h). These results show that the soluble N-terminal domain of FocA is necessary for the amide amino acid variant to efflux formate efficiently.

### Reduced growth of a strain synthesizing FocA_H209N_ is due to carbon loss

**Fig. 6.**
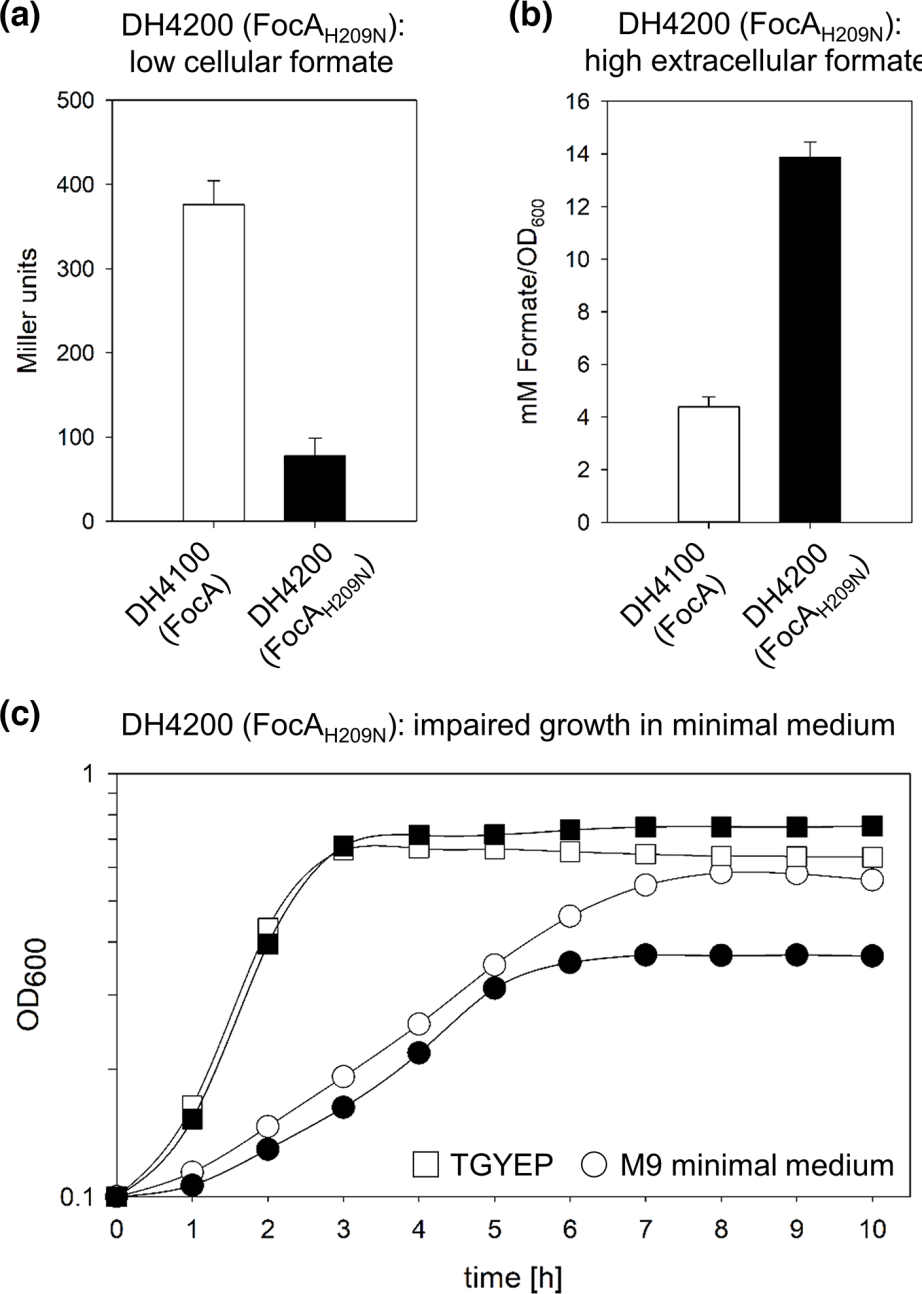
DH4200 (FocA_H209N_) is deficient in formate uptake and exhibits reduced anaerobic growth in minimal medium. Strain DH4200 (FocA_H209N_) was characterized with respect to both intra- and extracellular formate levels and its growth phenotype. (a) β-Galactosidase enzyme activity of DH4100 and DH4200 was analysed for cells grown anaerobically in glucose-M9-minimal medium. (b) The concentration of formate in the culture medium was determined by HPLC analysis. (c) The respective strains, DH4100 (closed symbols) and DH4200 (open symbols), were grown anaerobically in glucose-M9-minimal medium (circles) and rich medium TGYEP (squares), both supplemented with 0.8 % (w/v) glucose as carbon source. All experiments were performed with minimally three biological replicates, with each assay performed in duplicate.

It is evident that the final cell density attained by strain DH701 (*focA*) carrying plasmid pfocAH209N was ~35 % lower (Fig. 3c) than the same strain transformed with pfocA bearing the native *focA* gene (see e.g. Fig. 3a). This reduced growth could be due to either carbon limitation (e.g. loss of formate) or ion loss, e.g. ion leakage through the pore of the FocA_H209N_ variant. To distinguish between these possibilities, we exchanged the codon 209 of *focA* on the genome of strain DH4100 for an asparagine codon, delivering strain DH4200 (see Table S1 and Methods). First, we determined *fdhF_P_::lacZ* expression of the strain after anaerobic growth to he exponential phase (Fig. 6a) and observed a similar phenotype in terms of β-galactosidase enzyme activity to that for strain DH701 transformed with pfocAH209N (compare with Fig. 2b). Moreover, increased levels of formate were excreted into the growth medium by strain DH4200, relative to the levels excreted by DH4100 (synthesizing native FocA) (Fig. 6b). The final optical density attained after fermentative growth of strain DH4200 in minimal-glucose medium synthesizing chromosomally encoded FocA_H209N_ was significantly reduced compared with the parental strain DH4100 (Fig. 6c). This difference in growth between the two strains was, however, alleviated by cultivation of the strain in rich medium (Fig. 6c). Aerobic growth of strains DH4200 and DH4100 in minimal-glucose medium was indistinguishable (Fig. S4), which is consistent with lack of *focA* gene expression in the presence of oxygen [6]. Together, these findings suggest that the reduced growth of strain DH4200 might be due to carbon (CO_2_) loss through formate efflux by FocA_H209N_.

### Native FocA exhibits phenotypic dominance over FocA_H209N_

**Fig. 7.**
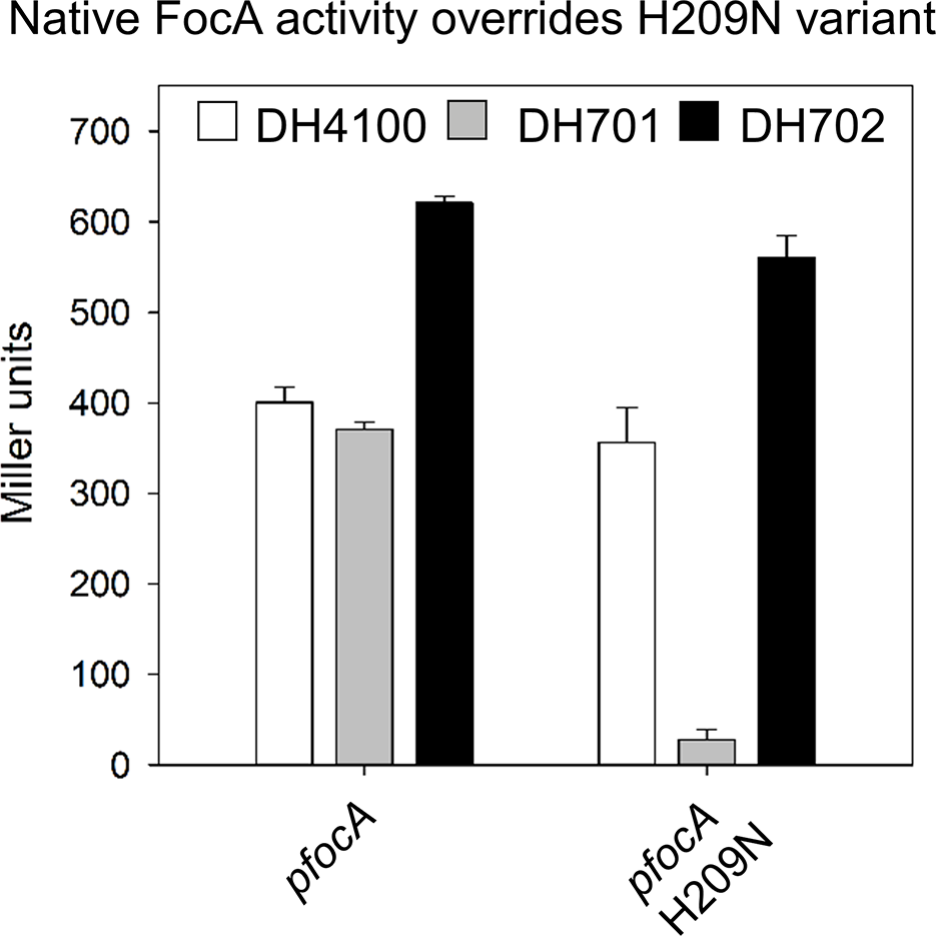
Native FocA shows dominance over the N209 exchange variant. Determination of β-galactosidase enzyme activity after anaerobic growth of the wild-type strain DH4100 (black histogram), the *focA* mutant DH701 (no FocA synthesized, grey histogram) and strain DH702 (10-fold elevated FocA levels; white histogram) transformed with a plasmid carrying a gene encoding either native FocA or FocA_H209N_. The strains were grown anaerobically in glucose-M9-minimal medium. The experiment was performed with minimally three biological replicates.

All the data presented so far indicate that the FocA_H209N_ and FocA_H209Q_ variants function exclusively as formate efflux channels. Because the vast majority of FNT-family members have a histidine residue within their translocation pore (Fig. 1b), this suggests strong selective pressure maintains histidine at this position. To provide evidence in support of this notion, we introduced plasmid pfocAH209N into the parental strain DH4100, as well as into the *focA* mutant DH701. In agreement with previous findings, measurement of β-galactosidase enzyme activity revealed that in DH701 synthesizing FocA_H209N_ β-galactosidase activity was barely detectable (Fig. 7). When FocA_H209N_ was synthesized in strain DH4100, additionally synthesizing chromosomally encoded native FocA, β-galactosidase enzyme activity was ≥13 fold higher, and in the range of that determined for native FocA synthesized in this genetic background (Fig. 7). To confirm this apparent dominance effect of native FocA over FocA_H209N_, we also introduced pfocAH209N into strain DH702 (Table S1), which produces approximately 10-fold more chromosomally encoded native FocA [6]. A similarly high level of β-galactosidase enzyme activity to that in DH4100 was measured, indicating that the strong formate efflux activity of FocA_H209N_ was overcome by the presence of native FocA in the same cell (Fig. 7). Taken together, these results indicate that chromosomally encoded, native FocA with a histidine residue within the pore overrides the efflux activity of FocA_H209N_ and re-imports formate to a concentration sufficient to induce expression of the *fdhF_P_::lacZ* gene fusion. This further supports our contention that H209 within the pore of FocA is essential for formate uptake.

## Discussion

### The histidine residue in the translocation pore is required for formate uptake by FocA

We show in this study that the centrally located H209 residue within the pore of FocA is essential for efficient, pH-dependent formate uptake into the *E. coli* cell, but is dispensable for formate efflux. This suggests that the protonated imidazolium cation of H209 is required for uptake of formate. This was demonstrated by exchanging the central H209 for a non-protonatable amide amino acid residue, generating an exclusive formate efflux channel, which was not competent to import formate or its chemical analogue hypophosphite. A previous study showed that exchange of H209 with a tyrosine residue caused intracellular formate accumulation [21], suggesting that exchanging H209 with a large, uncharged aromatic side-chain sterically blocks the pore, impeding formate efflux. Nevertheless, by exchanging the side-chain of histidine with that of a smaller, amide side-chain our results demonstrate that the histidine residue at position 209 is not essential for formate efflux and indicates that the mechanisms of formate efflux and influx through FocA are different, despite using the same pore.

Our findings also reconcile aspects of previous mechanistic proposals with respect to the dielectric environment facilitating anion passage [22], and with respect to protonation of H209 possibly transiently transferring the proton to formate [17, 18, 37]. Either of these proposals could theoretically explain passage of formate through the hydrophobic core of FocA. Clearly, however, we establish that H209 does not only have a structural role within the pore, as previously proposed [19, 22]. Rather, our *in vivo* data demonstrate that formate or hypophosphite uptake by native FocA only occurs when histidine (and presumably the imidazolium cation) is present. Whether the proposal that the H209–T91–H_2_O network allows H^+^ recycling to allow passage of the anion through the hydrophobic core of the channel [17, 18] cannot be concluded based on the current study, but will require further experimental analysis.

From a physiological perspective, formate:proton symport would not be energetically beneficial to a fermenting bacterium, due to the dissipation of *pmf*. Although mechanistically challenging, it is conceivable that the strongly positively charged imidazolium side-chain in the hydrophobic environment of the pore [37] might be capable of drawing the formate (and hypophosphite) anion through the pore by coulombic attraction. This would not require H^+^-recycling, but would nevertheless necessitate protonation of the histidine residue and a mechanism for further translocation of the anion into the cytoplasm. This latter might involve the cytoplasmically localized N-terminal domain of FocA, which interacts with PflB [20, 30], possibly inducing a conformational change aiding release of formate into the cytoplasm. This hypothesis needs to be tested in future studies.

### FocA_H209N_ synthesis prevents hydrogen production

The H_2_-negative phenotype of DH701/pfocAH209N and DH4200 arises most likely because the strain does not make an FHL complex [5]. Expression of the *fdhF* and *hyc* genes, which encode the structural components of the FHL complex [2, 3, 5], is induced only when formate is present at low millimolar concentrations within the cell [1, 5]. This, in turn, is because the formate-responsive transcription factor, FhlA, has an apparent *K*_m_ for formate of 5 mM [35]. Assuming FocA_H209N_ functions as an anion channel [38], invoking Nernstian behaviour [39], and a membrane potential difference of approximately −140 mV [40], then if the external formate concentration is 20 mM, the intracellular concentration of formate will never exceed the low micromolar range, i.e. several orders of magnitude below that required to be sensed by FhlA. The efficacy of FocA_H209N_ in anion efflux is exemplified by the fact that even with a p*K_a_* of 3.75 for formate, and adding a further 20 mM exogenous formate, this still failed to result in induction of *fdhF* expression (Fig. 2b). This is markedly different for the histidine-bearing (native) FocA channel, which, together with an induced and active FHL complex, enables the fermenting cell to exert much greater fine control of intracellular formate levels.

### Is formate an important reservoir of CO_2_ for anaerobic *E. coli* cells?

We made the unexpected observation that anaerobic growth of a strain synthesizing the FocA_H209N_ variant (e.g. strain DH4200) grew significantly more poorly than either the parental strain DH4100 or the *focA* mutant, DH701. This growth deficiency of DH4200 could, however, be circumvented by cultivation of the strain in rich medium. While this growth recovery in rich medium suggests that loss of cellular carbon might be the cause of the phenotype, it is also conceivable that this effect is the result of an imbalance in membrane potential due to excessive anion loss. Moreover, formate is not known to be utilized by wild-type *E. coli* as a growth substrate [3, 41]; however, it might be able to augment levels of CO_2_ necessary for heterotrophic CO_2_ fixation. It is known that CO_2_ released by the activity of decarboxylases is re-utilized by cellular carboxylases, explaining why mutants lacking carbonic anhydrase can only grow under high partial pressure of CO_2_ [42]. Nevertheless, an unequivocal explanation for the growth defect of strain DH4200 will require further experimentation.

### The question of FocA mechanism and the bioenergetics of fermentation

A further advantage of the controlled efflux and influx of formate in native FocA would be that, even if uptake of formate is in symport with that of H^+^, the production of gaseous H_2_ would effectively remove this proton from the cytoplasm, balancing *pmf* through the possibility of periplasmic re-oxidation of H_2_ [1–3]. This is energetically beneficial for the cell even though the FHL complex (Hyc-based components) cannot additionally pump protons [2], especially if FocA influxes formate and not formic acid. Importantly, in the efflux direction, due to the fact that H209 is dispensable for this function, it is likely that formate:H^+^ co-translocation occurs, thus contributing to *pmf*, which also supports the proposal that this occurs when the histidine is not protonated. This is based on the fact that the hydrophobicity of the core of the FocA pore would not permit ion passage [37, 43]. This would be in analogy to what has been demonstrated for lactate:proton symport in the lactic acid bacteria [44]. The formate regulon [5] thus combines intracellular formate-sensing, formate-regulated gene expression, formate disproportionation and ultimately substrate translocation to poise exquisitely the utilization of this valuable electron donor and carbon source [1–3]. Understanding how FocA controls cellular formate levels also helps to explain how formate-dependent ‘adaptive’ enzyme synthesis works, as originally proposed by Stephenson and Stickland [45].

### What is the explanation for the 1 % of FNTs that escape selective pressure to maintain a histidine residue?

In the comparatively few examples of naturally occurring FNT channels that have an amide amino acid residue in place of the central histidine, it can be proposed that these channels have evolved to act as unidirectional anion efflux channels. Examples include NirC-like nitrite channels in the anammox-utilizing *Planctomycetes* [46], a lactate channel in the protozoan pathogen *Entamoeba histolytica* [19] and FNT channels of unknown anion specificity in certain pathogenic firmicutes. The prevalence of FNT channels with a central histidine in the pore seems to be due to the selective advantage afforded by pH-controlled, bidirectional anion translocation. Generally, the broad distribution of FNT channels in anaerobes suggests an ancient evolutionary origin for these membrane proteins [7]. The structural and mechanistic parallels between FNTs and aqua- and glyceroporins [47, 48], including the key role of a protonatable histidine residue within the translocation pore [49], supports the view that these channels may represent a remarkable example of convergent evolution.

## Supplementary Data

Supplementary material 1
