# Supplementary material for: A single amino acid exchange converts FocA into a unidirectional efflux channel for formate
Source: Microbiology (Reading). 2022 Jan 27;168(1):001132. doi: 10.1099/mic.0.001132 (PMC8914244; doi:10.1099/mic.0.001132)
Supplement: Supplementary material 1 [file mic-168-1132-s001.pdf]

A Single Amino Acid-Exchange Converts FocA into a Unidirectional Efflux Channel for Formate

Michelle Kammel[1], Oliver Trebbin[1§], Constanze Pinske[1], and R. Gary Sawers[1*]

[1]Institute for Biology/ Microbiology, Martin Luther University Halle-Wittenberg, Kurt-Mothes-Str. 3, 06120 Halle (Saale), Germany

[§] Current address: IMD Laboratory Oderland GmbH, Am Kleistpark 1, Frankfurt (Oder), Germany

[*]Address correspondence to: RG Sawers (https://orcid.org/0000-0003-0862-2683)

gary.sawers@mikrobiologie.uni-halle.de

Table S1 Strains and plasmids used in this study

Strains and plasmids	Relevant genotype or characteristics	Reference or source
Strains		
BL21 (DE3)	F⁻ *ompT hsdS*(rB- mB-) *gal dcm lacY1* (DE3)	Invitrogen, Carlsbad, USA
MC4100	F⁻ *araD* Δ(*argF lac*) *U 169 ptsF25 deoC1 relA1 fblB530 rpsL 150 λ*⁻	[1]
MC4200	Like MC4100 but *focA* codon 209 changed to AAC	This study
REK701	Like MC4100 but *focA* (*focA* codons 114 and 115 changed to UAG and UAA)	[2]
REK702	Like MC4100 but *focA* (GUG codon of *focA* converted to AUG)	[2]
DH4100	MC4100 *λ(fdhF::lacZ)*	[3]
DH4200	MC4200 *λ(fdhF::lacZ)*	This study
DH601	MC4100 *focA* Δ*act* Δ(*act*::*cat* pACYC184) *λ(fdhFP::lacZ)*	[4]
DH701	REK701 *λ(fdhF::lacZ)*	[5]
DH702	REK702 *λ(fdhF::lacZ)*	This study
Phage		
λ(fdhF$_P$::lacZ)	λRS45 including the 232 bp regulatory region of *fdhF*	[3]
Plasmids		
pJET1.2	Amp^r, subcloning vector	Thermo Fischer Scientific, Waltham, USA
pMAK705	Cm^r, plasmid thermosensitive for DNA replication	[6]
pMAK705::*focA* H209N	pMAK705 carrying *focA* fragment with codon histidine 209 exchanged for asparagine	This study
pfocA3	Amp^r, expression vector with the gene *focA*	[5]
pfocA	Amp^r, expression vector with the gene *focA* (without StrepII tag)	This study
pfocAH209N	Amp^r, expression vector with gene *focA* (without StrepII tag), codon histidine 209 exchanged for asparagine	This study

pfocAH209Q	Amp^r, expression vector with gene *focA* (without StrepII tag), codon histidine 209 exchanged for glutamine	This study
pfocAΔK2-P31	Amp^r, expression vector with gene *focA* (without StrepII tag), Codons 2 to 31 are removed	[4]
pfocAH209N-ΔK2-P31	Amp^r, expression vector with gene *focA* (without StrepII tag), Codon 2 to 31 are removed and codon histidine 209 exchanged for asparagine	This study

Table S2 Oligonucleotide primers used in this study

Primers	Sequence 5’ → 3’[a]	Comment
focA_stop_fw	GAAAACGACCACCATTGAGCTTGG AGCCACCCG	Introduces a stop-codon at the 3´end of the *focA* gene
focA_stop_rev	CGGGTGGCTCCAAGCTCAATGGTGG TCGTTTTC	Introduces a stop-codon at the 3´end of the *focA* gene
focA_H209N_fw	CGGTTTTGAGAACAGTATCGCAAAC	Template for mutagenesis pfocA; [3]
focA_H209N_rev	GTTTGCGATACTGTTCTCAAAACCG	
focA_H209Q_fw	CGGTTTTGAGCAGAGTATCGCAAAC	Template for mutagenesis pfocA
focA_H209Q_rev	GTTTGCGATACTCTGCTCAAAACCG	
focA_pMAK_fw	GAGCTCGAAGGCCTACGAAAAGCT GC	Fragment for ligation into pMAK705 to construct the strain MC4200
focA_pMAK_rev	CAAGCTTGGTACGACCGAAGGACA TTG	

[a]Underlined bases indicate the substitution introduced in the *focA* gene

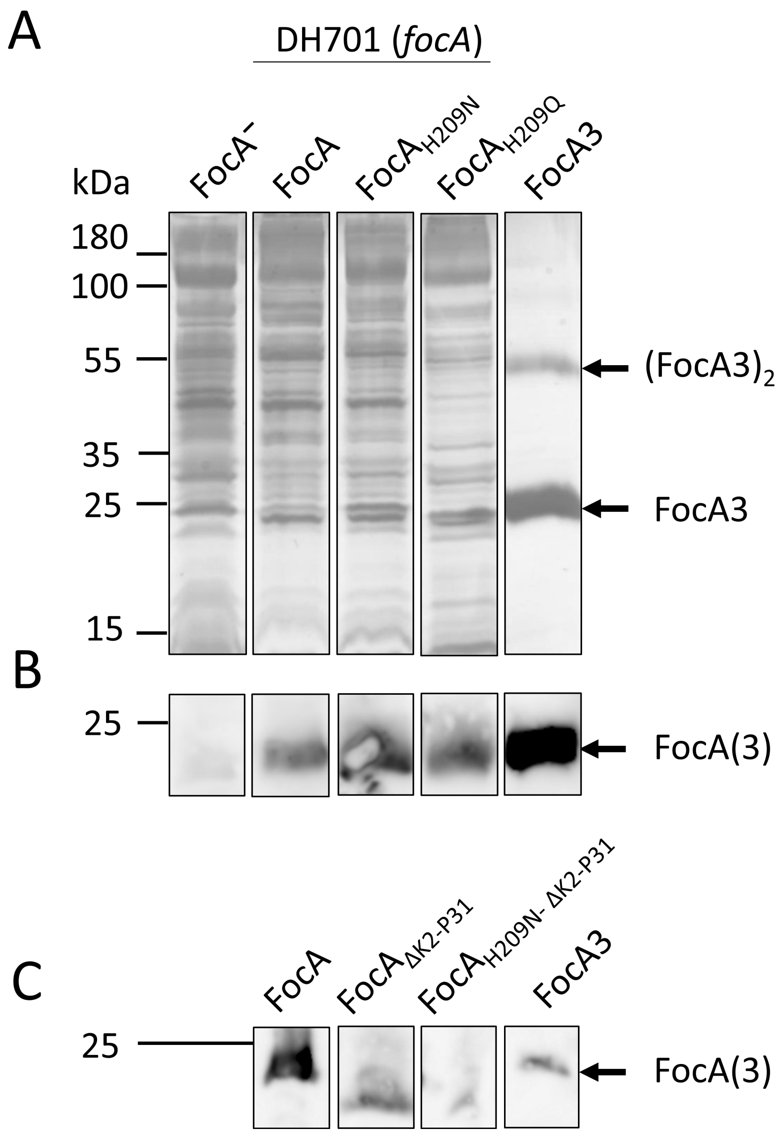

Figure S1. Synthesis of FocA (H209) and variants relative to native FocA.

Proteins in solubilised membrane fractions (50 µg protein A and B, 100 µg protein C) und 5 µg purified FocA (FocA3 with *C*-terminal StrepII-tag) were separated in a denaturing 12.5 % (w/v) polyacrylamide gel (A and B) or 16% Tris-tricine gel (C) and subsequently either silver-stained (A) or they were transferred to a nitrocellulose membrane and FocA was detected with affinity-purified antibodies raised against a FocA peptide (B and C) (see Methods in main text). The following strains were grown anaerobically in glucose-M9 minimal medium: FocA$^-$, DH701 (*focA*); DH701 transformed with plasmids carrying genes encoding the indicated FocA variants. In panel C, the medium used for cell growth included 0.2 µg ml^{-1} AHT. FocA3 signifies the purified recombinant protein with a *C*-terminal StrepII-tag. The migration positions of the molecular mass marker, PageRuler Prestained Protein Ladder (Thermo Fisher Scientific), are indicated in kDa on the left of the figure. The arrows indicate the monomer form of FocA3 and the dimer $(FocA3)_2$.

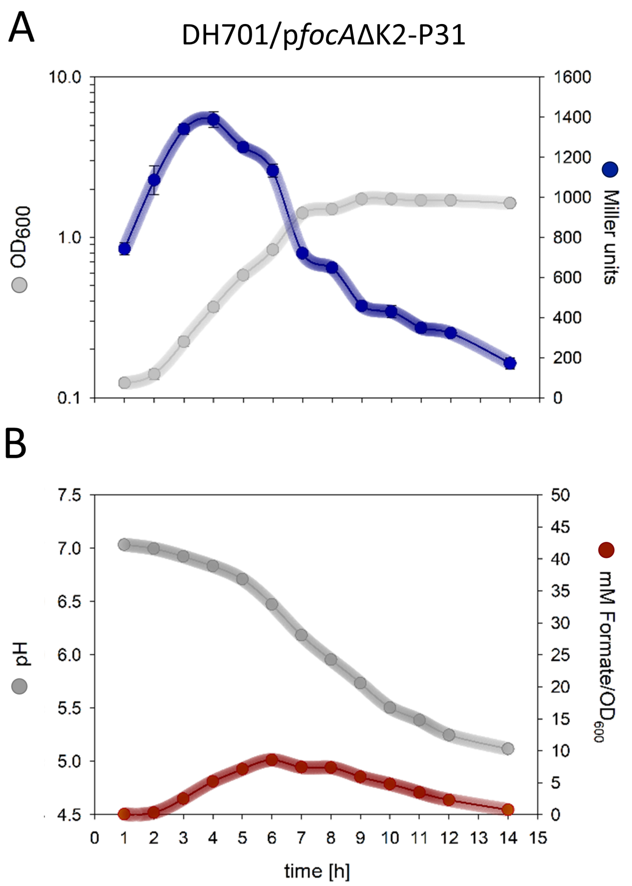

Figure S2. FocAΔK2-P31 works as bidirectional formate channel.

The strain DH701/pfocAΔK2-P31 was grown anaerobically in glucose-M9-minimal medium in crimp-sealed serum bottles. Samples were taken every hour to analyse optical density (OD_{600}) (A), determine β-galactosidase enzyme activity (A), pH (B) and formate levels in the culture medium (B). The colour-coding is as follows: light grey, OD_{600}; blue, β-galactosidase enzyme activity; dark grey, pH; and red, external formate concentration, which was calculated with respect to OD_{600}. All experiments were performed using three biological replicates.

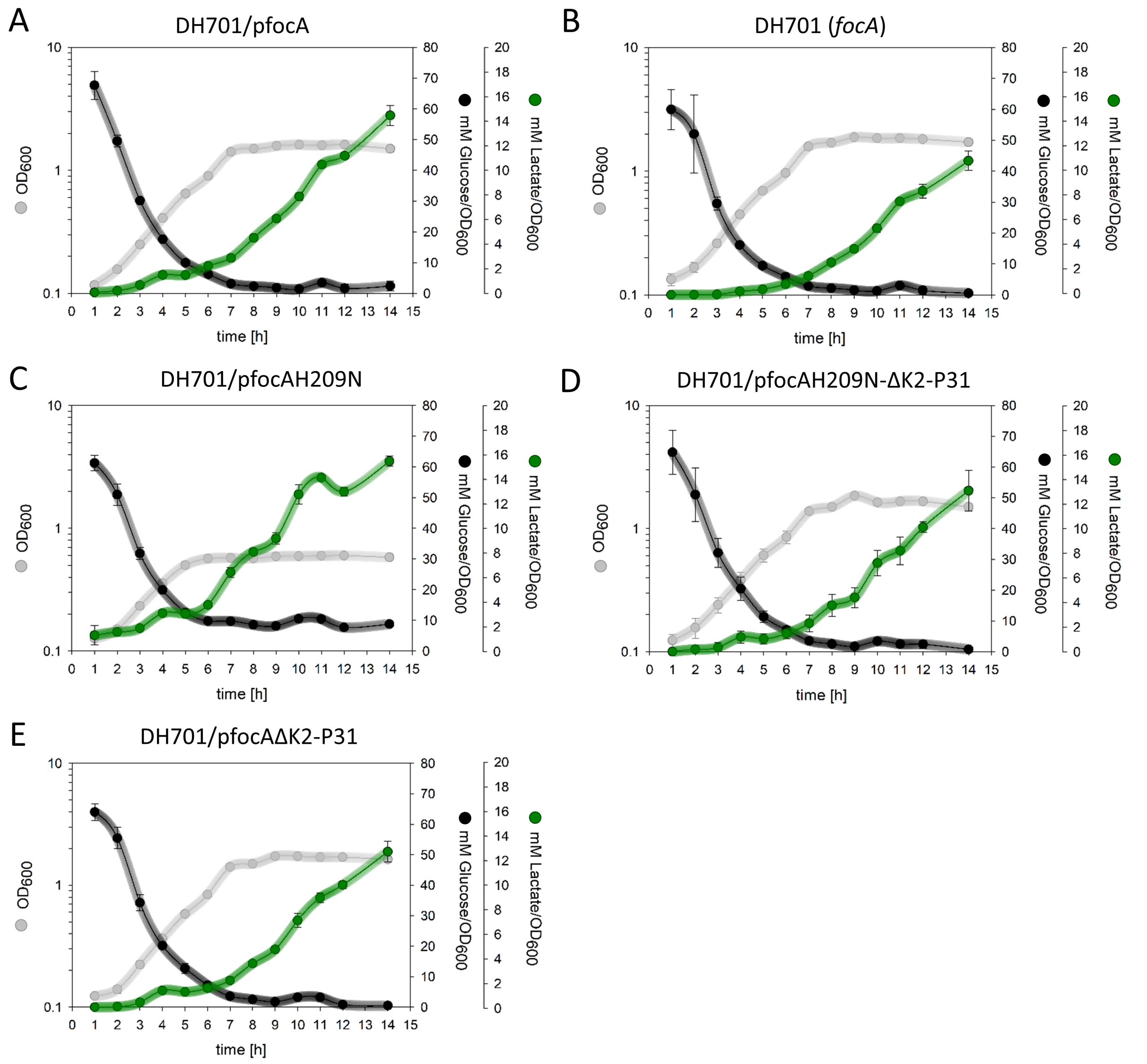

Figure S3. Glucose consumption and lactate production during anaerobic fermentation. Strains were grown anaerobically in glucose-M9-minimal medium. Samples were taken every hour to analyse optical density (OD_{600}) and to measure glucose and lactate levels in the culture medium. Panels show data for DH701/focA (A), DH701 (*focA*) (B), DH701/pfocAH209N (C), DH701/pfocAH209N-ΔK2-P31 (D) and DH701/pfocAΔK2-P31 (E). The colour-coding is as follows: light grey, OD_{600}; black, external glucose; and green, external lactate. Glucose and lactate concentrations were calculated with respect to OD_{600}. All experiments were performed using three biological replicates.

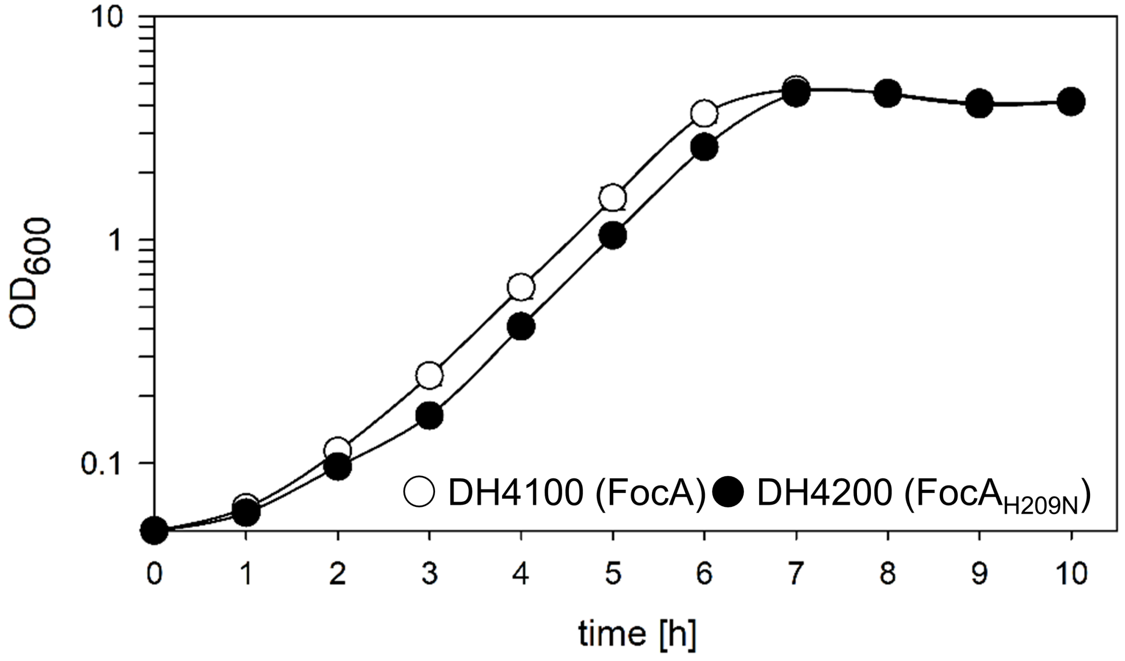

Figure S4. Strains DH4100 and DH4200 ($FocA_{H209N}$) have similar aerobic growth phenotypes.

The strains DH4100 (wild type) and DH4200 ($FocA_{H209N}$) were grown aerobically using shaking flasks in M9-minimal medium with 0.4 % (w/v) glucose. Samples were taken every hour to measure the optical density (OD_{600}). The growth analysis was performed with three biological replicates.

References

1. Casadaban MJ. Transposition and fusion of the *lac* genes to selected promoters in *Escherichia coli* using bacteriophage lambda and Mu. *J Mol Biol* 1976;104:541–555.
2. Suppmann B, Sawers G. Isolation and characterisation of hypophosphite-resistant mutants of *Escherichia coli*: identification of the FocA protein, encoded by the *pfl* operon, as a putative formate transporter. *Mol Microbiol* 1994;11965-982.
3. Hunger D, Doberenz C, Sawers RG. Identification of key residues in the formate channel FocA that control import and export of formate. *Biol Chem* 2014;395:813-825.
4. Kammel M, Hunger D, Sawers RG. The soluble cytoplasmic N-terminal domain of the FocA channel gates bidirectional formate translocation determinant. *Mol Microbiol* 2021;115:758-773.
5. Falke D, Schulz K, Doberenz C, Beyer L, Lilie H, *et al.* Unexpected oligomeric structure of the FocA formate channel of *Escherichia coli*: a paradigm for the formate-nitrite transporter family of integral membrane proteins. *FEMS Microbiol Letts* 2010;303:69-75.
6. Hamilton CM, Aldea M, Washburn BK, Babitzke P, Kushner SR. New method for generating deletions and gene replacements in *Escherichia coli J Bacteriol* 1989;171:4617-4622.
